# Clinical and echocardiographic benefit of Sacubitril/Valsartan in a real-world population with HF with reduced ejection fraction

**DOI:** 10.1038/s41598-020-63801-2

**Published:** 2020-04-20

**Authors:** Maria Vincenza Polito, Angelo Silverio, Antonella Rispoli, Gennaro Vitulano, Federica D’ Auria, Elena De Angelis, Francesco Loria, Alberto Gigantino, Domenico Bonadies, Rodolfo Citro, Albino Carrizzo, Gennaro Galasso, Guido Iaccarino, Carmine Vecchione, Michele Ciccarelli

**Affiliations:** 1 Chair of Cardiology, Department of Medicine, Surgery and Dentistry, Schola Medica Salernitana, University of Salerno, Salerno, Italy; 2 Department of Cardiology, A.O.U. “San Giovanni di Dio e Ruggi D’Aragona”, Salerno, Italy; 3 Vascular Pathophysiology Unit, IRCCS Neuromed, Pozzilli Isernia, Italy; 4 Department of Advanced Biomedical Sciences, “Federico II” University, Naples, Italy

**Keywords:** Cardiology, Outcomes research

## Abstract

The aim of this study was to evaluate the effects of Sacubitril/Valsartan (S/V) on clinical, laboratory and echocardiographic parameters and outcomes in a real-world population with heart failure with reduced ejection fraction (HFrEF). This was a prospective observational study enrolling patients with HFrEF undergoing treatment with S/V. The primary outcome was the composite of cardiac death and HF rehospitalization at 12 months follow-up; secondary outcomes were all-cause death, cardiac death and the occurrence of rehospitalization for worsening HF. The clinical outcome was compared with a retrospective cohort of 90 HFrEF patients treated with standard medical therapy. The study included 90 patients (66.1 ± 11.7 years) treated with S/V. The adjusted regression analysis showed a significantly lower risk for the primary outcome (HR:0.31; 95%CI, 0.11–0.83; p = 0.019) and for HF rehospitalization (HR:0.27; 95%CI, 0.08–0.94; p = 0.039) in S/V patients as compared to the control group. A significant improvement in NYHA class, left ventricular ejection fraction, left ventricular end systolic volume and systolic pulmonary arterial pressure was observed up to 6 months. S/V did not affect negatively renal function and was associated with a significantly lower dose of furosemide dose prescribed at 6- and 12-month follow-up. In this study, S/V reduced the risk of HF rehospitalization and cardiac death at 1 year in patients with HFrEF. S/V improved NYHA class, echocardiographic parameters and need of furosemide, and preserved renal function.

## Introduction

Despite the improvements in clinical management and medical therapy of heart failure (HF), the outcome of patients with HF and reduced ejection fraction (HFrEF) remains poor^1^. If compared to the other HF entities, this category shows distinct demographic characteristics, comorbidities, response to therapy, and a substantially higher risk of mortality secondary to sudden cardiac death (SCD) and rehospitalization for worsening HF^2^. Many HFrEF patients are still undertreated and several drugs, such as beta-blockers and ACE inhibitors (ACEi), are under-dosed. This issue is also related to the incorrect risk evaluation by clinicians, who are likely to consider the absence or paucity of symptoms as an indicator of HF stability^3^. In fact, HFrEF is a progressive disorder that, despite the improvement of patient’s clinical status achieved with conventional medications, may be associated with a high risk of adverse events at short and long-term follow-up^4^.

Current European guidelines^5^ recommend the use Sacubitril/Valsartan (S/V), an angiotensin receptor-neprilysin inhibitor (ARNI), in replacement of the renin–angiotensin–aldosterone system (RAAS) inhibition in ambulatory HFrEF patients still symptomatic despite optimal medical therapy (OMT). This recommendation comes from a single randomized study named PARADIGM-HF trial^6^, which showed the superiority of S/V compared to Enalapril in reducing the incidence of cardiovascular death or hospitalizations for HF. Few studies have so far investigated the benefit of S/V therapy in HFrEF patients treated in everyday clinical practice. Observational studies have the power to validate the results of randomized controlled trial, to identify areas in which investigation is needed and to test new hypotheses.

The present was designed as an observational, prospective study to monitor the impact of Sacubitril/Valsartan on clinical, laboratory, echocardiographic parameters as well as on clinical outcomes in a real-world HFrEF population.

## Results

### Study population

Ten patients developed side effects related to ARNI administration (symptomatic hypotension in 5 cases, worsening renal function in 2 cases, systolic blood pressure ≤95 mmHg in 2 cases, hyperkalemia in 1 case) and were excluded from the study. Finally, 90 patients were included in the study population for the analysis.

The baseline characteristics of the study population are reported in Table 1. Mean age was 66.1 ± 11.7 years and the majority of patients were males (78, 86.7%). Hypertension was reported in 81.1% of cases and ischemic etiology of HF in 63.3%.

**Table 1:** Baseline characteristics of Sacubitril/Valsartan and standard therapy groups (N = 180).

Variables	Sacubitril/Valsartan (N = 90)	Standard therapy (N = 90)	P-value
Age, yrs	66.1 ± 11.7	67.0 ± 11.2	0.590
Male sex, N (%)	78 (86.7)	80 (88.9)	0.649
BMI, kg/m^2^	27.6 (25.2–31.2)	27.1 (23.7–30.1)	0.161
BSA, m^2^	1.9 ± 0.2	1.9 ± 0.2	0.201
Systolic arterial pressure, mmHg	130.0 (120.0–140.0)	125.0 (120.0–135.0)	0.047
Diastolic arterial pressure, mmHg	80.0 (75.0–85.0)	80.0 (80.0–85.0)	0.876
Heart rate, bpm	70.0 (64.0–81.0)	69.0 (64.0–77.0)	0.559
Hypertension, N (%)	82 (91.1)	51 (56.7)	<0.001
Dyslipidemia, N (%)	70 (77.8)	63 (70.0)	0.235
Diabetes, N (%)	29 (32.2)	35 (38.9)	0.350
Ischemic etiology, N (%)	57 (63.3)	60 (66.7)	0.639
Smoking, N (%)	24 (26.7)	33 (36.7)	0.149
ICD, N (%)	31 (34.4)	46 (51.1)	0.024
CRT, N (%)	34 (37.8)	12 (13.3)	<0.001
CKD, N (%)	52 (57.8)	49 (54.4)	0.652
GFR, mL/min	56.0 (42.0–76.0)	59.0 (50.0–76.8)	0.223
Atrial fibrillation, N (%)	30 (33.3)	22 (24.7)	0.204
COPD, N (%)	25 (27.8)	21 (23.3)	0.494
Anemia, N (%)	30 (33.3)	25 (27.8)	0.418
Hb, g/mL	13.1 ± 2.3	12.7 ± 1.7	0.335
NYHA II, N (%)	47 (52.2)	40 (44.4)	0.296
NYHA III, N (%)	43 (47.8)	50 (55.6)	0.296
BNP, pg/Ml	488.0 (294.0–997.0)	547.0 (221.5–1200.0)	0.712
**Echocardiographic parameters**			
LVEF, %	31.0 (27.2–37.0)	31.5 (27.0–35.0)	0.588
LVEDV, mL	175.0 (142.3–221.8)	188.0 (147.8–227.0)	0.564
LVESV, mL	115.0 (93.0–152.8)	120.0 (96.5–166.0)	0.478
sPAP	31.0 (25.0–45.0)	35.0 (25.0–45.0)	0.491
TAPSE	19.2 ± 4.9	17.8 ± 4.3	0.062
E/e’	12.0 (10.0–18.0)	14.0 (12.5–16.0)	0.109
**Concomitant medications**			
Loop diuretics, N (%)	84 (93.3)	83 (92.2)	0.773
Beta-blockers, N (%)	83 (92.2)	85 (94.4)	0.550
ACE inhibitors, N (%)	—	76 (84.4)	—
ARBs, N(%)	—	14 (15.6)	—
Statins, N (%)	61 (67.8)	55 (61.1)	0.350
MRA, N (%)	46 (51.1)	52 (57.8)	0.369
Amiodarone, N (%)	19 (21.1)	20 (22.2)	0.856
Digoxin, N (%)	9 (10)	14 (15.6)	0.264
Ivabradine, N (%)	16 (17.8)	13 (14.4)	0.543

Continuous normally distributed variables are expressed as mean ± SD. Categorical variables are n (%). Continuous non-normally distributed variables are median (interquartile range).

ACE, angiotensin converting enzyme; ARBs, angiotensin receptor blockers; BMI, body mass index; BSA, body surface area; CKD, chronic kidney disease; COPD, chronic obstructive pulmonary disease; CRT, cardiac resynchronization therapy; E/e’, ratio of mitral E peak velocity and averaged e’ velocity; GFR, glomerular filtration rate; ICD, implantable cardioverter-defibrillator; NYHA, New York Heart Association functional class; BNP, brain natriuretic peptide; Hb, hemoglobin; eGFR, estimated glomerular filtration rate; LVEF, left ventricular ejection fraction; LVESV, left ventricular end systolic volume; LVEDV, left ventricular end diastolic volume; sPAP, pulmonary arterial systolic pressure; TAPSE, tricuspid annular plane systolic excursion; MRA, mineralocorticoid receptor antagonist.

At the time of recruitment, all patients were in NYHA class II (52.2%) or III (47.8%). CKD was reported in 57.8% of cases and the median baseline eGFR value was 58.5 (IQR 47.5–81.0) mL/min.

The study population showed an high risk profile as suggested by the low LVEF (31.0%, IQR 27.2–37.0) and the high LVEDV (175.0 mL, IQR 142.3–221.8) and LVESV (115.0 mL, IQR 93.0–152.8). Loop diuretics were prescribed in 93.3% of cases, MRA in 51.1%, beta blockers in 92.2%, ivabradine in 17.8% and statins in 67.8%.

### Clinical outcome at follow-up

**Figure 1:**
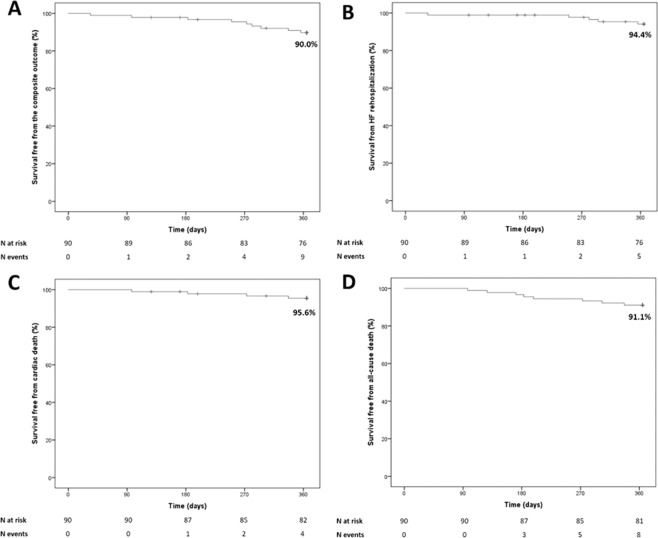
Survival free from worsening HF (panel A), composite outcome (panel B), cardiac death (panel C) and all-cause death (panel D) in the study population. HF, heart failure.

One-year survival free from the study clinical outcomes in the study population is reported in Fig. 1. No patient was lost at follow-up. The Kaplan-Meier estimate for the primary outcome at 1 year was 90.0%; the estimates for rehospitalization for worsening HF, cardiac death and all-cause death were 94.4%, 95.6% and 91.1%, respectively.

**Figure 2:**
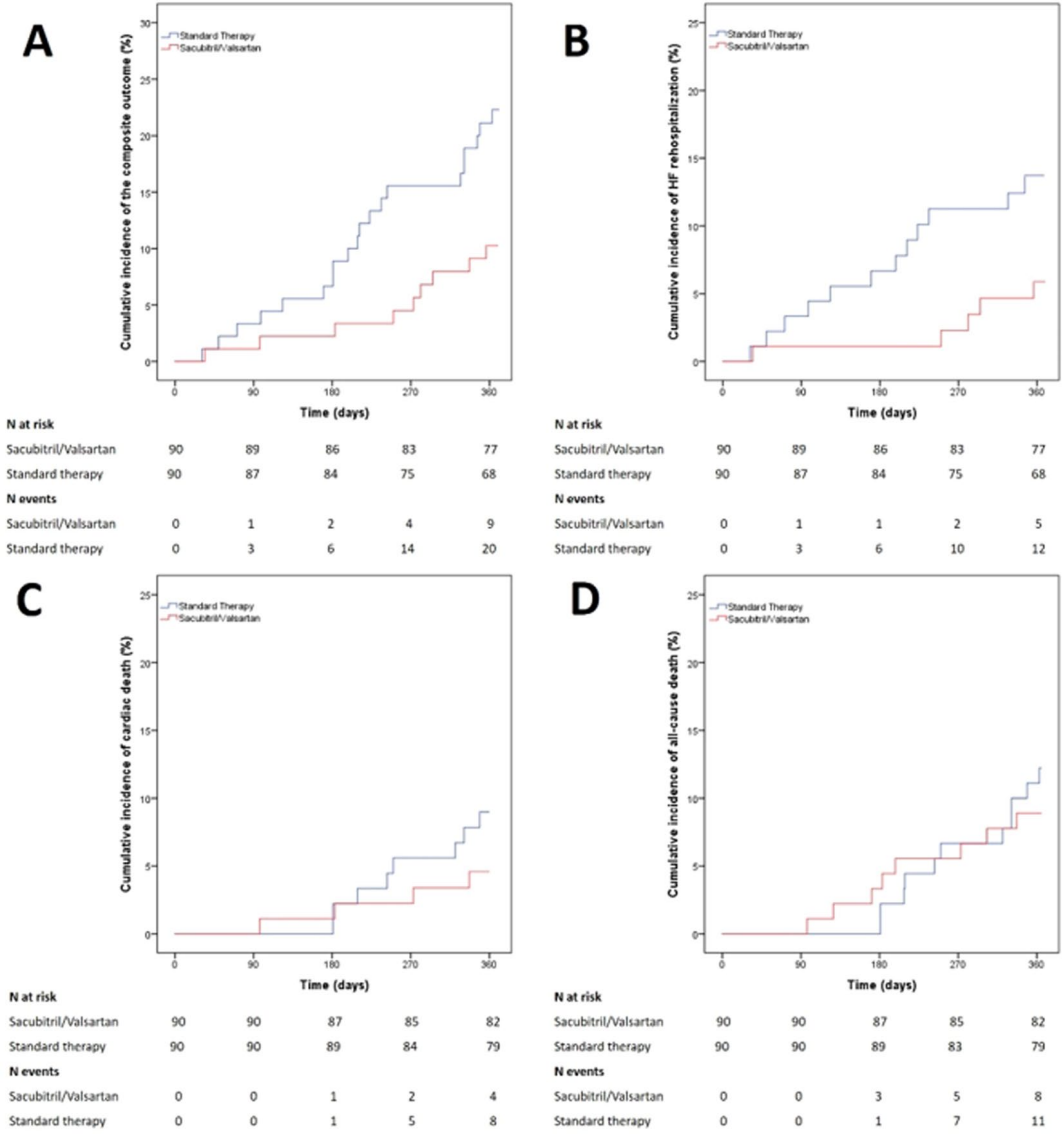
Cumulative incidence of rehospitalization for worsening HF (panel A), composite outcome (panel B), cardiac death (panel C) and all-cause death (panel D) in Sacubitril/Valsartan and standard therapy groups HF, heart failure.

The cumulative incidence of events in the S/V as compared to the standard therapy group is shown in Fig. 2. The adjusted and unadjusted HR for all the study outcomes are reported in the Table 2. The propensity score weighting adjusted regression analysis showed a significantly lower risk for HF rehospitalization (HR: 0.27; 95%CI, 0.08–0.94; p = 0.039) and for the composite outcome (HR: 0.31; 95%CI, 0.11–0.83; p = 0.019) in patients treated with S/V as compared to the control. No differences were observed for the risk of cardiac death (HR: 0.50; 95%CI, 0.09–2.66; p = 0.413) and all-cause death (HR: 0.84; 95%CI, 0.26–2.72; p = 0.769) between groups.

**Table 2:** Adjusted and unadjusted HR for the study outcomes.

Study outcomes	Unadjusted model		
	HR	95% CI	p-value
HF rehospitalization	0.40	0.14–1.12	0.081
Cardiac death	0.50	0.15–1.67	0.262
Composite outcome	0.42	0.19–0.93	0.032
All-cause death	0.73	0.30–1.82	0.503
	**Adjusted model**		
	HR	95% CI	p-value
HF rehospitalization	0.27	0.08–0.94	0.039
Cardiac death	0.50	0.09–2.66	0.413
Composite outcome	0.31	0.11–0.83	0.019
All-cause death	0.84	0.26–2.72	0.769

CI, confidence interval; HF, heart failure; HR, hazard ratio.

### Effects of S/V on the symptoms, LV remodeling, and eGFR at 6 months follow-up

**Figure 3:**
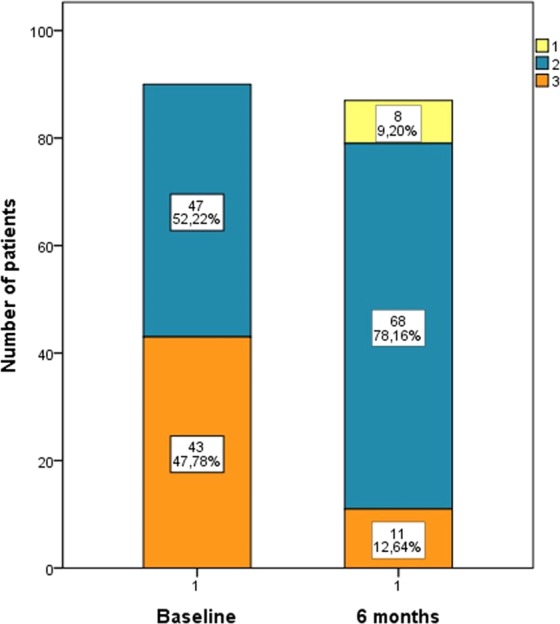
Bar graphs showing the changes of NYHA class from baseline to 6 months in patients treated with Sacubitril/Valsartan.

The changes of NYHA class from baseline to 6 months follow-up are summarized in Fig. 3. Noteworthy, S/V was associated with a substantial improvement of NYHA class up to 6 months (NYHA II: from 52.2% to 78.2%; NYHA III: from 47.8% to 12.6%). Of note, eight patients (9.2%) reached NYHA class I.

**Figure 4:**
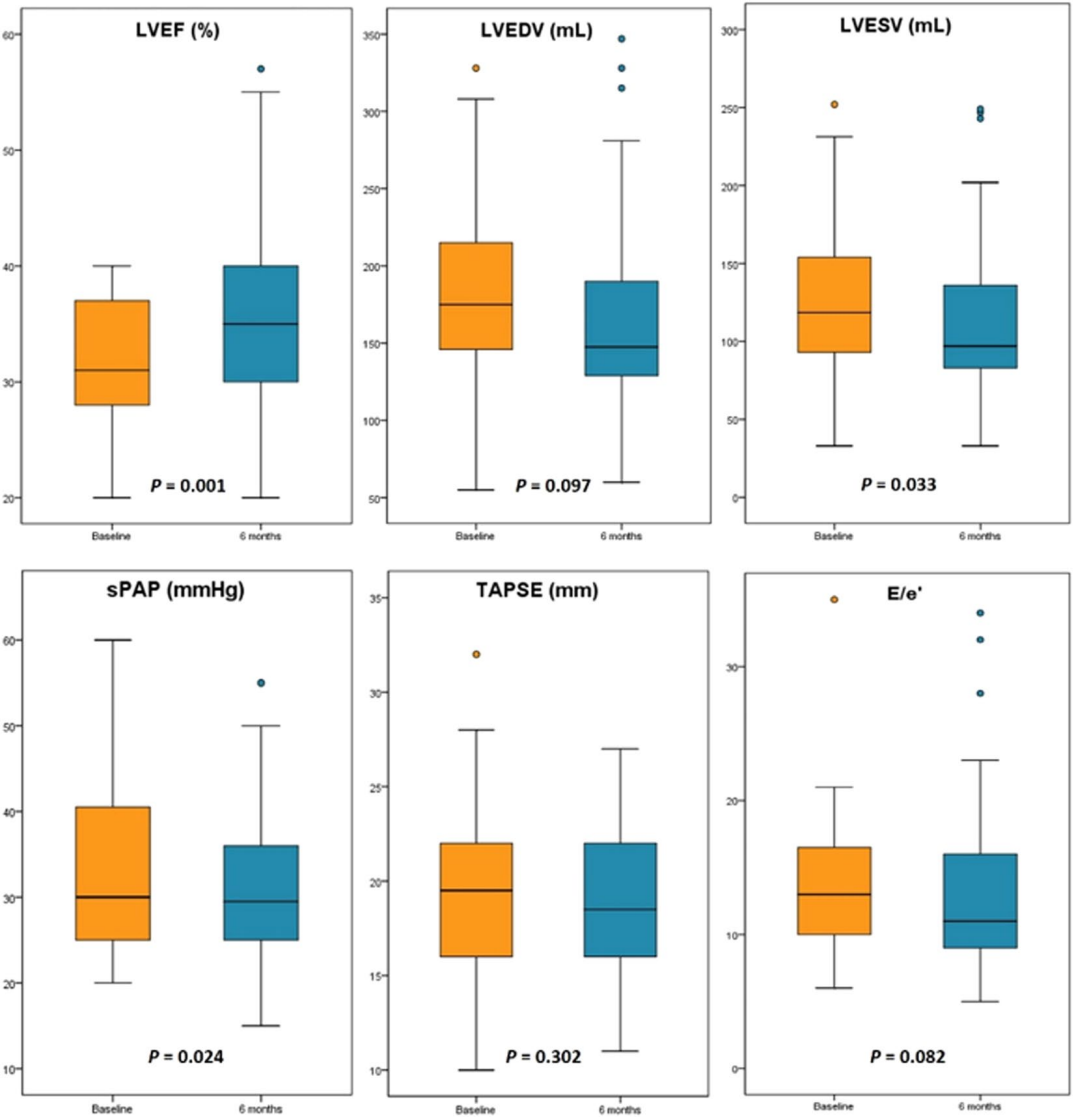
Box plots showing the changes of echocardiographic parameters form baseline to 6-months follow-up in patients treated with Sacubitril/Valsartan. The boxes represent the interquartile range of the samples and include the median value (horizontal line). The line across the boxes reache the maximum and minimum values. LVEF, left ventricular ejection fraction; LVEDV, left ventricular end diastolic volume; LVESV, left ventricular end systolic volume; sPAP, systolic pulmonary artery pressure; TAPSE, tricuspid annular plane systolic excursion.

LVEF significantly increased from 31.0% (IQR 27.2–37.0) to 34.0% (IQR 29.2–39.7; p = 0.001**;** Fig. 4). As well, LVESV significantly decreased from 115.0 mL (IQR 93.0–152.8) to 101.0 mL (IQR 83.5–141.5; p = 0.033) and sPAP decreased from 31.0 mmHg (IQR 25.0–45.0) to 25 mmHg (IQR 25–34; p = 0.024). A substantial, albeit not statistically significant, improvement in LVEDV, TAPSE and E/e’ values was achieved.

**Figure 5:**
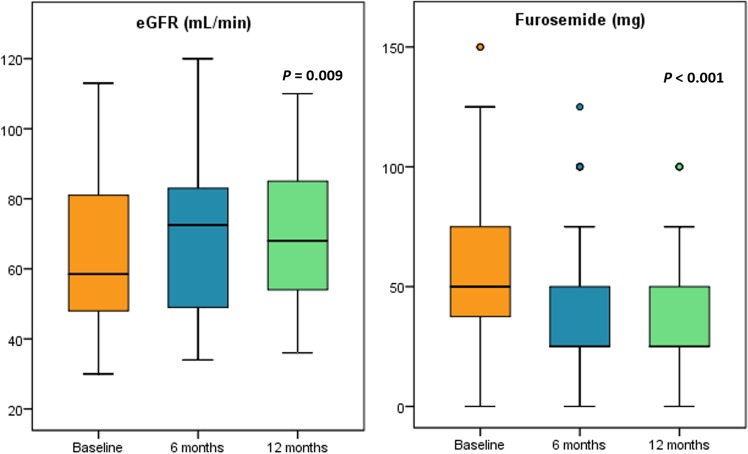
Box plots showing the changes in term of eGFR and furosemide dose regimen from baseline to 6- and 12-months follow-up in patients treated with Sacubitril/Valsartan. eGRF, estimated glomerular filtration rate.

Furthermore, we observed a significant improvement of eGFR from 56.0 mL/min (IQR 42.0–76.0) to 72.5 mL/min (IQR 48.7–83.7) at 6-months and 68.0 mL/min (IQR 53.5–86.0) at 12-months follow-up (p = 0.009; Fig. 5). A significantly lower dose of furosemide prescribed [50 mg (IQR 37.5–100.0) at baseline, 25 mg (IQR 25.0–56.3) at 6 months and 25 mg (IQR 25.0–50.0) at 12 months; p < 0.001)] was reported during the follow-up (Fig. 5).

## Discussion

The present study focused on the impact of S/V in a real-world HFrEF population. The main findings can be summarized as follows: (i) S/V reduces the risk of the composite outcome and of new hospitalization for HF worsening at 12 months follow-up in comparison with the standard medical therapy; (ii) S/V is associated with a significant improvement in NYHA class, LVEF, LVESV, and sPAP at 6 months follow-up: (iii) eGFR improved and furosemide dose was lower in patients treated with S/V at 6 months follow-up.

HFrEF population is characterized by distinct clinical features^7^ and a higher risk of SCD and HF rehospitalization than other HF cohorts^8,9^. Thus, HFrEF patients need to be carefully evaluated in order to stratify prognosis and identify higher-risk subjects requiring a close clinical follow-up and frequent drug and dose adjustment^10^. Current HF guidelines recommend a stepwise medical approach based on clinical symptoms, which includes the use of S/V in place of ACE-I or ARBs in ambulatory HFrEF patients who remain symptomatic despite optimal medical treatment^5^. Noteworthy, 40% HF patients in NYHA class II die of SCD also without worsening symptoms^3^. Probably, this may be related mainly to a misperception of symptom that leads the physician to underestimate the risk and do not optimize the treatment. Therefore, HF care should not be based primarily on only symptoms and, perhaps, it is licit to affirm that it is always the appropriate moment to optimize HF therapy. However, we lack of evidences regarding the impact of S/V on HF symptoms, LV remodeling, renal function and clinical outcome in real-world HFrEF populations^11^. The paucity of data on the safety and effectiveness of S/V in the real-world practice represent a current critical issue.

Our population showed a similar age and prevalence of comorbidities than the PARADIGM-HF trial, suggesting that the patients randomized in this study had characteristics similar to the general population with HFrEF treated in the real world^12^. In term of clinical outcome, our observational findings are consistent with the results of the PARADIGM-HF trial, which showed a significant reduction of 21% in the rate of HF rehospitalizations. We also found a numerical but not statistical difference in term of cardiac death between S/V and standard therapy groups. This result may be related to the small sample size of our population and to the relatively short (1-year) follow-up time. These results should be interpreted also in light of the dosage of S/V adopted in our population, since only 17 of 90 patients received the maximal dose of 97 mg/103 mg. This datum is consistent with previous evidence from TITRATION trial, which reported an overall tolerability of the maximum dose in 76% of patients^13^, and confirm that also lower dose of S/V is associated to a clinical benefit as compared to standard medical therapy with conventional RAAS system inhibitors^14^.

In a previous observational study by Norberg *et al*.^15^ only a quarter of the patients with HFrEF fulfilled the inclusion criteria of the PARADIGM-HF trial. Conversely, we prospectively enrolled a selected population according to the above mentioned criteria, which have been embed in the current HF guidelines^5^.

Rodil Fraile *et al*.^16^ have recently demonstrated that S/V relieves symptoms and improves functional NYHA class in HFrEF patients with multiple pathologies and advanced HF (NYHA III/IV at baseline). As in our study, a significant improvement in the NYHA class was recorded at follow-up. In comparison to PARADIGM trial, our patients treated with S/V were more symptomatic at baseline, as suggested by NYHA class III recorded in greater percentage, and showed a statistically significant improvement of symptoms at follow-up.

Interestingly, in addition to the improvement of HF symptoms, we have found a positive effect of S/V on echocardiographic parameters up to 6 months.

LV reverse remodeling is clearly an outcome of interest in HF patients since it is associated with a poor outcome at follow-up^17^. Thus, the improvement of LV volume and function is a goal of clinical care^18^ in order to prevent the progression of HF and improve outcome.

To date, only one smaller retrospective study enrolling 48 HFrEF patients^19^ treated with S/V has showed a significant improvement in LVEF, LV mass and diameters at 3 months median follow-up. In out cohort, we confirm the improvement in LVEF along with a significant reduction of LVESV and sPAP up to 6 months. Of note, S/V was able to reduce sPAP values despite the reduction in the use of furosemide, suggesting a role of the LV reverse remodeling in the improvement of pulmonary hemodynamics.

In experimental HFrEF models, it has been demonstrated that S/V improved cardiac function by reducing myocardial fibrosis, prevented cardiac rupture after myocardial infarction (MI) by inhibiting the inflammation and the degradation response of macrophages, and improved survival and LV volumes after MI if compared to enalapril^20^.

Our study seems to support the safety of S/V in terms of renal function in HFrEF patients. In HFpEF patients, ARNI therapy was previously associated with significantly greater preservation of eGFR when compared with valsartan therapy alone^21^. Moreover, in the PARADIGM trial the rate of discontinuation for renal impairment was lower in the S/V than in the enalapril group (0.7% vs. 1.4% respectively, p = 0.002). The blockade of the RAAS system is a well-studied approach in preventing chronic renal injury and slowing the progression of CKD^22^. In animal models, also natriuretic peptides (NPs) have shown antioxidant, anti-inflammatory and antifibrotic proprieties and vasodilator action on the afferent arteriole which may contribute to renoprotective effects of S/V. Our results are consistent with previous studies showing that S/V led to a slower rate of decrease in the eGFR, even in patients with CKD, compared with ACEIs/ARBs therapy^23^. However, the observational nature of our study and the statistical analysis based on an “as treated” approach (S/V was early discontinued in two patients due to worsening renal function) do not allow definite conclusions, and further studies are needed to confirm our findings.

These results may also be confirmed by the lower dose of furosemide prescribed in patients treated with S/V.

In HFrEF, the dose of diuretics should be as low as possible to reach and maintain euvolemia and to prevent adverse effect related to electrolyte disorders. Many studies on HFrEF patients^24,25^ have shown that higher doses of loop diuretics were associated with worse clinical outcome and this association was most pronounced for the risk of HF rehospitalizations. Therefore, a reduction of dosage at follow-up represent a surrogate endpoint suggesting a good course of the disease.

## Study limitation

The small sample size and the single-center study design may affect the generalizability of our results. Moreover, our observations could not be applied to patients with HFmEF or HFpEF as well as to patients with HFrEF who do not meet the PARADIGM-HF entry criteria^26^.

Despite we performed a propensity score weighting analysis in order to adjust for baseline patient-related variables between patients treated with S/V or standard therapy, we cannot exclude a residual bias secondary to other concealed confounders.

Since we enrolled patients in HF outpatient clinic setting, patients with NYHA functional class IV were not included in the study. Moreover, paired eGFR data between baseline and 6 months follow-up were available only in 38 patients. Therefore, the results on renal function need to be confirmed by further larger researches.

## Conclusions

In a real-world HFrEF population, S/V reduced significantly the occurrence of the composite of cardiac death and rehospitalizations for worsening HF up to 1 year and improved NYHA class, LV EF, LVESV and sPAP at 6 months follow-up. Furthermore, S/V did not negatively affect renal function and reduced the need of furosemide at 6 months follow-up.

## Methods

### Study population, clinical outcome and follow-up

For this study we applied the ESC Guidelines for the diagnosis and treatment of acute and chronic heart failure^5^. Specifically, from June 2016, date of the first prescription of S/V in our dedicated HF outpatient clinic, to December 2017 one hundred patients with chronic HFrEF diagnosed by echocardiography [left ventricular ejection fraction (LVEF) ≤ 40%] and treated with S/V were prospectively evaluated. The protocol was established according to the ethical guidelines of the Declaration of Helsinki and informed consent was obtained from all participants. All patients were in AHA/ACC Stage C and NYHA Class II/III. At the time of enrollment, the eligibility to therapy with S/V was evaluated according to the PARADIGM-HF inclusion and exclusion criteria^6^. The dose of ARNI was progressively increased up to the maximally tolerated dose according to current guidelines^5^. Regular measurements of systolic blood pressure and periodic determinations of electrolytes (particularly serum potassium and sodium), creatinine were prescribed. The estimated glomerular filtration rate (eGFR) was calculated by using the Chronic Kidney Disease Epidemiology Collaboration equation^27^. Patients previously treated with RAAS system inhibitors, withheld ACEi or Angiotensin II Receptor Blockers (ARBs) a day before the initiation of treatment with S/V, in order to minimize the risk of angioedema. The decision to maintain an intermediate dose (49 mg/51 mg twice daily) was reached in 37 patients and to reduce the dose to 24 mg/26 mg twice daily in 28 patients. Only in 17 patients, the dose was increased until 97 mg/103 mg tablet twice a day (**Supplementary Material-Online** Table 1). Conversely, in all the remaining cases, a dosage reduction of S/V was planned if the association at the higher dose was not tolerated. Beta-blockers (carvedilol, bisoprolol, or nebivolol), ivabradine, mineralocorticoid receptor antagonists (MRA; spironolactone, eplerenone or canrenoate), furosemide, amiodarone, digoxin were prescribed as recommended by current guidelines^5^ and according to the patient clinical status.

All baseline features were prospectively collected on a predefined computerized data sheet, including demographic, clinical, laboratory, electrocardiographic and echocardiographic data. Clinical follow-up was periodically performed in our HF outpatient clinic. Clinical outcome was assessed in all patients up to 12 months. The primary clinical outcome was the occurrence of composite of cardiac death and HF rehospitalization. Other secondary clinical outcomes were: all-cause death, cardiac death and rehospitalization for worsening HF.

Echocardiographic parameter were collected at baseline and up to 6 months (LVEF, LVEDV, LVESV, sPAP, TAPSE, E/e’). Furosemide dose and eGFR data at baseline, 6 months and 12 months were also collected.

To estimate the clinical impact of S/V with respect to the standard HF therapy, the study population was compared with a retrospective HFrEF cohort of 90 patients treated in our HF outpatient clinic between January 2014 to May 2016 (before introduction of ARNI in clinical practice). The comparator group consisted of patients treated with the standard HF drugs (including ACEIs or ARBs) up-titrated to the maximum tolerated dose, as recommended by the guidelines^28^, and maintained for at least 3 months.

### Transthoracic echocardiography

Standardized transthoracic echocardiography (TTE) was performed following the American Society of Echocardiography (ASE) and European Association of Cardiovascular Imaging (EACVI) guidelines^29^. Echocardiographic exams were performed using a commercially available system (Vivid E9; GE-Vingmed Ultrasound, Horten, Norway) and a 3.5-MHz transducer. All parameters were analyzed offline by two expert operators blinded to clinical data. The echocardiographic analysis included the evaluation of left ventricular end-diastolic (LVEDV) and end-systolic (LVESV) volumes. LV systolic function was assessed by determining LVEF with biplane analysis using the modified Simpson’s rule. For the assessment of early-diastolic filling (E) the pulsed-wave Doppler sample volume was positioned at the tip of the tenting area of the mitral valve in the apical long axis view. Mean e’ was assessed in the basal inferoseptal and lateral LV region in the apical 4-chamber view using Tissue Doppler Imaging^30^. The ratio of mitral E peak velocity and averaged e’ velocity (E/e’) was calculated. As a parameter of the global right ventricular function, tricuspid annular plane systolic excursion (TAPSE) was assessed by aligning the M-mode linear cursor to the lateral tricuspid annulus in the apical four-chamber view. Systolic pulmonary artery pressure (sPAP) was derived from the tricuspid regurgitant jet velocity using systolic trans-tricuspid pressure gradient calculated by the modified Bernoulli equation and the addition of estimated right atrial pressure according to inferior vena cava dimension and inspiratory collapsibility.

### Statistical analysis

Distribution of continuous data was tested with the Kolmogorov–Smirnov and the Shapiro-Wilk test. Normally distributed variables were expressed as mean ± standard deviation (SD) and compared using the Student t test, whereas non-normally distributed ones as median and interquartile range (IQR) and compared with the Mann-Whitney U test. Categorical variables were reported as numbers and percentages and compared using the χ2 or Fisher exact tests, as appropriate^31^. Paired samples t-test was performed to assess changes in continuous echocardiographic data between baseline and 6 months follow-up. Repeated measures analysis of variance (ANOVA) test was performed to assess changes in eGFR and furosemide data through baseline, 6 and 12 months follow-up. The cumulative incidence of HF rehospitalization, cardiac death, all-cause death and composite outcome was estimated at different time frames using the Kaplan-Meier method.

The unadjusted and adjusted hazard ratios for the clinical outcomes of interest were calculated using the Cox proportional hazard regression model and presented as hazard ratio (HR) with by their 95% confidence intervals (CI). We used propensity score weighting to account for potential selection bias in treatment assignment (S/V vs standard therapy) between the two groups (average treatment effect weights). The following pre-treatment covariates were included in the propensity score models: age, gender, body mass index, hypertension, dyslipidemia, diabetes, smoking, chronic kidney disease (CKD), atrial fibrillation, chronic obstructive pulmonary disease, anemia, ischemic etiology, implantable cardioverter-defibrillator, cardiac resynchronization therapy, New York Heart Association (NYHA) functional class, LVEF, loop diuretics, beta-blockers, statins. Standardized mean difference was calculated to assess the balance for all covariates included in the propensity score model (**Supplementary Material-Online** Fig. 1). For all test, a p values <0.05 was considered statistically significant. Statistical analysis was performed by using SPSS software version 23.0 (SPSS Inc., Chicago, Illinois) and R version 3.5.1 (R Foundation for Statistical Computing, Vienna, Austria).

### Statement on ethical approval and on informed consent

The authors confirm that all research was performed in accordance with relevant guidelines/regulations. This is an observational study performed in accordance with ESC Heart Failure Guidelines 2016. The study population was compared with a retrospective HFrEF cohort managed in accordance with ESC Heart Failure Guidelines 2012. Informed consent was obtained from all participants.

## Supplementary information

Supplementary information.
