# Supplementary material for: Clinical and echocardiographic benefit of Sacubitril/Valsartan in a real-world population with HF with reduced ejection fraction
Source: Sci Rep. 2020 Apr 20;10:6665. doi: 10.1038/s41598-020-63801-2 (PMC7170843; doi:10.1038/s41598-020-63801-2)
Supplement: Supplementary file 1 — Supplementary information. [file 41598_2020_63801_MOESM1_ESM.docx]

**Clinical and echocardiographic benefit of Sacubitril/Valsartan in a real-world population with HF with reduced ejection fraction**

Maria Vincenza Polito ^1^, Angelo Silverio ^1^, Antonella Rispoli ^1^, Gennaro Vitulano ^1^, Federica D’Auria ^1^, Elena De Angelis ^1^, Francesco Loria ^1^, Alberto Gigantino ^2^, Domenico Bonadies ^2^, Rodolfo Citro ^2^, Albino Carrizzo ^3^, Gennaro Galasso ^1^, Guido Iaccarino ^4^, Carmine Vecchione ^1^, Michele Ciccarelli ^1*^.

1. *Chair of Cardiology, Department of Medicine, Surgery and Dentistry, Schola Medica Salernitana, University of Salerno, Salerno, Italy*
2. *Department of Cardiology, A.O.U. “San Giovanni di Dio e Ruggi D'Aragona”, Salerno, Italy*
3. *Vascular Pathophysiology Unit, IRCCS Neuromed, Pozzilli, Isernia, Italy*
4. *Department of Advanced Biomedical Sciences, “Federico II” University, Naples, Italy*

**SUPPLEMENTARY MATERIAL**

**Online Table 1. Doses of Sacubitril/Valsartan in study population (N= 82*)**

| **Dose** | **N, %** |
| --- | --- |
| 24/26 mg twice daily | 37 (45.1) |
| 49/51 mg twice daily | 28 (34.2) |
| 97/103 mg twice daily | 17 (20.7) |

** 8 patients were excluded since dead at follow up*

**ONLINE FIGURE LEGEND**

**Online Figure 1**

**
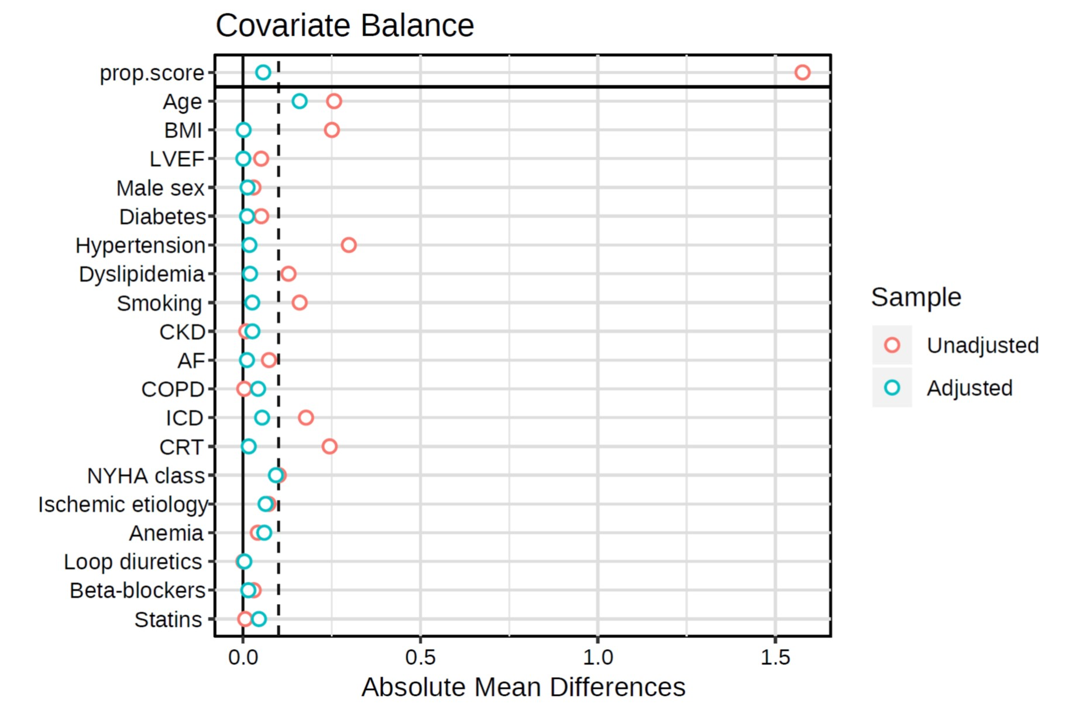
**

Love plot showing the unadjusted (red circles) and adjusted (blue circles) values of the absolute standardized difference before and after weighting. The dotted line represents the threshold value of 0.1 for the absolute standardized mean difference; standardized mean difference values <0.1 are conventionally considered an index of optimal balance achieved after weighting.

*BMI, body mass index; LVEF, left ventricular ejection fraction; CKD, chronic kidney disease; AF, atrial fibrillation; COPD, chronic obstructive pulmonary disease; ICD, implantable cardioverter-defibrillator; CRT, cardiac resynchronization therapy; NYHA, New York Heart Association functional class.*
